# Editorial: Towards 2030: sustainable development goal 3: good health and wellbeing. A sociological perspective

**DOI:** 10.3389/fsoc.2025.1616878

**Published:** 2025-06-17

**Authors:** Andrzej Klimczuk, Sangeeta Chattoo, Chimaraoke Izugbara, Magdalena Klimczuk-Kochańska, Piotr Toczyski, Ashwani Kumar, Rubal Kanozia, Grzegorz Piotr Gawron, Ahmad Ozair

**Affiliations:** ^1^Department of Social Policy, Collegium of Socio-Economics, SGH Warsaw School of Economics, Warsaw, Poland; ^2^Department of Sociology, University of York, York, United Kingdom; ^3^International Center for Research on Women, Washington, DC, United States; ^4^School of Public Health and Community Medicine, University of Gothenburg, Gothenburg, Sweden; ^5^Faculty of Management, University of Warsaw, Warsaw, Poland; ^6^Media and Communication Department, Institute of Philosophy and Sociology, The Maria Grzegorzewska University, Warsaw, Poland; ^7^Metagenomics and Secretomics Research Laboratory, Department of Botany, University of Allahabad, Prayagraj, India; ^8^Department of Mass Communication and Media Studies, School of Information and Communication Studies, Central University of Punjab, Bathinda, India; ^9^Institute of Sociology, Faculty of Social Sciences, University of Silesia in Katowice, Katowice, Poland; ^10^Faculty of Medical Sciences, King George's Medical University, Lucknow, India; ^11^Bloomberg School of Public Health, Johns Hopkins University, Baltimore, MD, United States

**Keywords:** SDG3, essential health services, public health, universal health coverage, health inequalities, wellbeing, global health

## Overview

This Research Topic explores the third Sustainable Development Goal (SDG) to “ensure healthy lives and promote wellbeing for all at all ages,” highlighting the severe impact of the COVID-19 pandemic. The pandemic has threatened to reverse prior improvements in maternal and child health, immunization coverage, and reductions in communicable and non-communicable disease mortality while also disrupting essential health services, shortening life expectancy, and worsening mental health issues and healthcare access inequalities. This Research Topic contains studies examining how social stratification, geography, and culture affect health, aiming to inform policymakers on developing equitable public health policies to address disparities and improve wellbeing.

The Research Topic “*Towards 2030: sustainable development goal 3: good health and wellbeing. A sociological perspective*” includes 11 articles prepared in total by 52 authors from the following countries: Brazil, Canada, Ethiopia, India, Norway, Spain, Sweden, the United Kingdom, and the United States. “Frontiers in Sociology” and “Frontiers in Public Health” journals were responsible for organizing this project. The presented Research covering: six original research articles (Tripathi and Samanta; Zewude et al.; Teteh et al.; Engdawork et al.; Kåks et al.; Elung'ata), two hypothesis and theory papers (Sæbø and Lund; Field-Richards and Timmons), one conceptual analysis (Coca et al.), one review (Araújo et al.), and one study protocol (Martí-Lluch et al.). The Research Topic covers topics such as aging, community support, social determinants of health, digital health, health promotion and prevention, health-related stigma, and social innovation. The articles comprising this Research Topic are divided into three themes.

## Theme I: advancements and challenges in health systems

The first paper of this Research Topic by Araújo et al. analyzes the alignment of Brazilian and international organizations' arguments for adopting digital health in primary health care during the COVID-19 pandemic to support achieving the SDGs. This study finds that both emphasize the applicability of information and communication technologies, but Brazil also underscores the need for regulatory frameworks to support these digital practices. Field-Richards and Timmons focus on specific cases of risk management related to cardiac arrest (CA) that lead to high mortality and morbidity, with many deaths in intensive care units due to neurological injury following out-of-hospital CA. Despite the uncertainty and risk in prognostication, post-cardiac arrest (P-CA) guidelines emphasize using prognosticators to manage professional risk, balancing the duty to prognosticate with the challenges of accurately predicting outcomes. Martí-Lluch et al.'s study argues that healthcare and wellbeing depend on multiple factors that must adapt to societal changes, including individuals' increasing participation in their care decisions. Their analysis focuses on the association between personal aptitudes related to behaviors and health outcomes. It contributes to improved health promotion and prevention strategies by examining their impact on morbidity, mortality, lifestyle adoption, quality of life, and healthcare utilization. Finally, Elung'ata's study examines healthcare systems as mesocosms to understand social disparities in spousal violence perceptions in sub-Saharan Africa, focusing on cohort differences in victim decision-making. Using data from 18 countries, it finds significant differences in spousal violence decisions and underlines the influence of healthcare system access, supporting socio-ecological theory in addressing these disparities.

## Theme II: social determinants and innovations in health and wellbeing

The subsequent papers mainly focus on factors and innovations influencing health and wellbeing. Tripathi and Samanta's study explores how leisure activities can moderate the relationship between subjective wellbeing and depressive symptoms among older Indians, using data from the Longitudinal Aging Study in India. Results indicate that increased social engagement through leisure activities significantly improves wellbeing and reduces depressive symptoms, highlighting the need for age-friendly initiatives and social infrastructure to enhance involvement and wellbeing in older adults. Kåks et al. studied the adaptation of a South African social innovation based on peer support for mothers that was implemented in southern Sweden to help immigrant women access public health services. The research found that the intervention's success relied on trust-building and flexible, tailored support despite community mistrust and funding issues. The social determinants of health (SDOH) are also crucial in Sæbø and Lund's study of smoker stigma, an unintended consequence of tobacco policies in Norway that is influenced by personal values and socio-demographic factors. Their research found high levels of perceived public stigma against smokers, especially among women, young people, and high socio-economic status individuals, with smokers planning to quit feeling the most stigma. In another study, Teteh et al. examined how SDOH influences the quality of life (QOL) outcomes for lung cancer surgery patients. Their research found that financial challenges, education, healthcare access, environment, and social support impact QOL, highlighting the need for routine SDOH assessment and targeted interventions to improve care and survivorship.

## Theme III: health-related strategies and interventions

The last section provides examples of specific case studies related to health-related public policies. Engdawork et al. focus on Ethiopia's response to the COVID-19 pandemic, which involved structural and social interventions, such as national policy development, community engagement, and mass communication strategies, which helped slow the virus spread in Addis Ababa. However, challenges such as competing interests, misconceptions, and capacity constraints hindered implementation, emphasizing the need for future interventions to address these issues in low- and middle-income settings. The team of Zewude et al. focused on Addis Ababa, particularly on the situation of street children, who are highly vulnerable to health risks and have limited access to healthcare services. This study found that while these children perceive their susceptibility to health risks and engage in preventive behaviors such as maintaining hygiene and physical exercise, they also frequently engage in risky behaviors such as smoking, sniffing glue, and unprotected sex, which necessitates targeted health interventions. Finally, Coca et al. examined the socio-historical importance of pharmacovigilance. The authors show that this scientific discipline has evolved significantly, as highlighted by the sulfonamide elixir case. The paper suggests that integrating pharmacovigilance with the social sciences could enhance its societal impact and promote a more democratic environment responsive to individual and group needs.

## Conclusion

The research findings presented in this Research Topic facilitate the formulation of five directions for further research combined with SDG3. These are: (1) transformations of social determinants of health, covering new lifestyles and smart environments (see Missoni, 2022; Ozair and Singh, 2021); (2) new schemes in the health emergency preparedness and access to universal health coverage (see Okyere et al., 2024); (3) public health and health sector innovations in the post-COVID-19 pandemic period (see Raji and Demehin, 2023); (4) integrated innovation combining ideas from the fields the environmental policy, social policy, and health policy (see Klimczuk et al., 2022); and (5) privacy and ethical issues related to e-health solutions such as telemedicine platforms, mobile health apps, and wearable health devices (see Wyllie et al., 2022).
